# Towards High-Energy and Anti-Self-Discharge Zn-Ion Hybrid Supercapacitors with New Understanding of the Electrochemistry

**DOI:** 10.1007/s40820-021-00625-3

**Published:** 2021-03-18

**Authors:** Yang Li, Wang Yang, Wu Yang, Ziqi Wang, Jianhua Rong, Guoxiu Wang, Chengjun Xu, Feiyu Kang, Liubing Dong

**Affiliations:** 1 College of Chemistry and Materials Science, Jinan University, Guangzhou, 511443 People’s Republic of China; 2 Centre for Clean Energy Technology, University of Technology Sydney, Sydney, NSW 2007 Australia; 3 Tsinghua Shenzhen International Graduate School, Tsinghua University, Shenzhen, 518055 People’s Republic of China

**Keywords:** Zn-ion hybrid supercapacitor, Carbon material, Fibrous cathode, Hierarchical pore structure, High-energy

## Abstract

Aqueous Zn-ion hybrid supercapacitors (ZHSs) are increasingly being studied as a novel electrochemical energy storage system with prominent electrochemical performance, high safety and low cost. Herein, high-energy and anti-self-discharge ZHSs are realized based on the fibrous carbon cathodes with hierarchically porous surface and O/N heteroatom functional groups. Hierarchically porous surface of the fabricated free-standing fibrous carbon cathodes not only provides abundant active sites for divalent ion storage, but also optimizes ion transport kinetics. Consequently, the cathodes show a high gravimetric capacity of 156 mAh g^−1^, superior rate capability (79 mAh g^−1^ with a very short charge/discharge time of 14 s) and exceptional cycling stability. Meanwhile, hierarchical pore structure and suitable surface functional groups of the cathodes endow ZHSs with a high energy density of 127 Wh kg^−1^, a high power density of 15.3 kW kg^−1^ and good anti-self-discharge performance. Mechanism investigation reveals that ZHS electrochemistry involves cation adsorption/desorption and Zn_4_SO_4_(OH)_6_·5H_2_O formation/dissolution at low voltage and anion adsorption/desorption at high voltage on carbon cathodes. The roles of these reactions in energy storage of ZHSs are elucidated. This work not only paves a way for high-performance cathode materials of ZHSs, but also provides a deeper understanding of ZHS electrochemistry.

**Supplementary Information:**

The online version contains supplementary material available at 10.1007/s40820-021-00625-3.

## Highlights

- A surface engineering strategy was proposed to design hierarchically porous structure on fibrous carbon cathodes with O/N heteroatom functional groups.
- High-energy and anti-self-discharge Zn-ion hybrid supercapacitors (ZHSs) were realized.
- ZHS electrochemistry was investigated and new insights were provided.

**Supplementary Information:**

The online version contains supplementary material available at 10.1007/s40820-021-00625-3.

## Graphical abstract

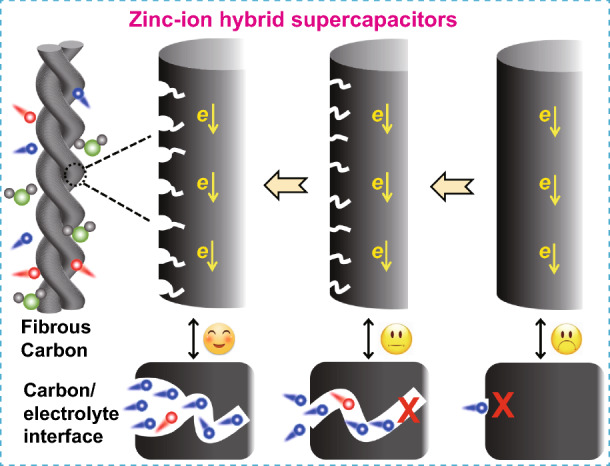

## Introduction

Electrochemical energy storage (EES) receives increasing attention benefiting from its prominent merits of high energy conversion/storage efficiency and low pollution. Rapid development of economic society puts forward higher requests to energy density, power density, safety and service lifetime of electrochemical energy storage systems, and consequently, various electrochemical energy storage systems such as lithium-ion batteries, potassium-ion batteries, sodium-ion batteries, multivalent-ion (e.g., Zn^2+^, Mg^2+^, Ca^2+^, and Al^3+^) batteries and supercapacitors have been developed [1–7]. Metal-ion batteries with organic electrolytes generally possess high working voltage and high energy density, whereas their power density, security performance and lifespan are not satisfactory in comparison with supercapacitors, but unfortunately, supercapacitors especially aqueous ones always suffer from low energy density which is associated with their intrinsic charge-storage mechanism (e.g., charge accumulation at electrode/electrolyte interface) and low working voltage [1, 8]. As another type of electrochemical energy storage systems, hybrid supercapacitors are composed of one battery-like electrode that can provide high energy density and one capacitor-like electrode that can provide high power density and good cycling stability. Therefore, hybrid supercapacitors theoretically combine the advantages of batteries and supercapacitors, and thus gain much attention [1, 9–13].

Zn-ion hybrid supercapacitors (ZHSs) are a novel electrochemical energy storage system [14–17]. In particular, since we proposed an aqueous ZHS system with high specific surface area activated carbon material (AC) cathode, metallic zinc anode, and ZnSO_4_ aqueous electrolyte, attractive features including high energy density, high power density, ultralong cycle life, and superior safety of ZHSs became apparent [14]. The superior performance of the AC//Zn ZHS originates from fast ion adsorption/desorption on cathode surface, high capacity (823 mAh g^−1^) and low redox potential (− 0.76 V vs. standard hydrogen electrode, SHE) of zinc anode, as well as utilization of aqueous electrolyte. In recent years, aqueous ZHSs are increasingly regarded as a promising electrochemical energy storage system, and many efforts have been made to synthesize high-performance cathode materials, such as hydrous RuO_2_ [18], graphene nanosheets [19, 20], polymer or biomass derived carbon materials [21–25], oxidized carbon nanotubes [26], MXene nanoflakes [27], phosphorene [28], and so on [29–31]. Among them, porous carbon materials are outstanding candidates for ZHS cathodes that can achieve relatively high capacity (> 100 mAh g^−1^), due to their high specific surface area and developed pore structure. This seems to be similar to the ion storage behaviors of carbon electrodes in H_2_SO_4_, Na_2_SO_4_ and KOH electrolytes [1]. However, different from univalent cations such as H^+^, Na^+^, and K^+^, each Zn^2+^ cation carries two charges and has a larger hydrated radius of 0.430 nm, impelling us to re-design carbon pore structure to efficiently accommodate divalent Zn^2+^ cations (actually, anion storage should also be taken into account). This is an important basis in realizing high-capacity and high-rate carbon cathodes. It should also be noted that self-discharge behavior has a significant impact on the practical application of electrochemical energy storage devices, but carbon-based aqueous supercapacitors commonly suffer serious self-discharge, which is caused by charge redistribution inside carbon pores, carbon oxidization and so on [32, 33]. Pore structure and surface functional groups of carbon electrodes always affect their self-discharge behaviors [33–36]. The use of porous carbon cathodes will possibly bring self-discharge problem to carbon//Zn ZHSs, and then expunge ZHSs’ advantage of relatively high working voltage. Therefore, regulating surface physicochemical characteristics of carbon cathodes to realize anti-self-discharge ZHSs is a meaningful topic. In addition, electrochemical energy storage mechanism of carbon//Zn ZHSs is not well elucidated yet. For instance, some researches consider Zn^2+^ cation adsorption/desorption on carbon cathodes, but ignore anion storage behaviors and their effects on electrochemical properties of ZHS cathodes. Meanwhile, basic zinc sulfate nanoflakes with chemical formula of Zn_4_SO_4_(OH)_6_·*n*H_2_O are always observed on carbon cathodes during charge/discharge processes of carbon//Zn ZHSs [14, 22, 37, 38], but their formation is difficult to understand merely based on simple ion adsorption/desorption theory on carbon surface. Since currently reported carbon cathodes are generally composed of carbon active materials, conductive additives, binder and metal current collectors, existence of the latter three components affects phase identification of carbon cathodes at different charge/discharge states, thus hindering energy storage mechanism investigation of ZHS cathodes. Besides, introduction of conductive additives, binder and metal current collectors also notably lowers gravimetric capacity and energy density of ZHS devices.

Herein, we designed hierarchically porous structure on fibrous carbon surface with O/N heteroatom functional groups and realized high-energy and anti-self-discharge ZHSs. For the fabricated fibrous carbon materials, their surface physicochemical characteristics and electrochemical properties when utilized as free-standing cathodes of ZHSs were comprehensively studied, and also, roles of hierarchical pore structure and heteroatom functional groups during energy storage process of the fibrous carbon cathodes were discussed in detail. Furthermore, to reveal energy storage mechanism of carbon cathodes and understand ZHS electrochemistry, electrochemical analysis of cation/anion storage behaviors and phase identification of reaction products during charge/discharge processes of the fibrous carbon cathodes-based ZHSs were performed.

## Experimental Section

### Preparation of Fibrous Carbon Cathodes

Activated carbon fiber product (Nantong Senyou Carbon Fiber Co., Ltd.) was produced through a steam-activation method. It was washed using deionized water and dried at 80 °C in our laboratory, and used as micropore-dominated carbon fiber material (denoted as “MPCF”). MPCF was soaked in a sufficient amount of 5 M KOH aqueous solution for 24 h and thoroughly dried at 60 °C in a vacuum oven. Then, the MPCF containing KOH was heat-treated at 850 °C for 1 h in N_2_ atmosphere with a heating rate of 5 °C min^−1^, washed with deionized water and dried at 80 °C for 12 h. The fabricated sample was hierarchically porous carbon fiber material and denoted as HPCF.

### Assembly and Electrochemical Measurements of ZHSs

Commercial carbon fiber, MPCF and HPCF samples were directly used as free-standing cathodes for ZHSs, which means that conductive additives (e.g., acetylene black) and binder were not needed. ZHSs in the form of CR2032 coin cells were assembled with the above fibrous carbon cathodes, zinc foil anode (50 μm in thickness and 12 mm in diameter), air-laid paper separator and 2 M ZnSO_4_ or 2 M Zn(CF_3_SO_3_)_2_ aqueous electrolyte. Electrochemical properties of the ZHSs were evaluated after 6 h since their assembly. Cyclic voltammetry (CV) tests at scan rates of 2–100 mV s^−1^ were carried out on a Bio-logic VMP3 electrochemical workstation. Galvanostatic charge–discharge (GCD) measurements at low current densities of 0.1–1 A g^−1^ were performed on a LAND battery-testing instrument, while GCD measurements at relatively large current densities of 2–20 A g^−1^ were performed on the Bio-logic VMP3 electrochemical workstation, because electrochemical workstation has higher precision for fast charge/discharge tests. Calculation formulas of specific capacity, energy density and power density were provided in Supporting Information. For electrochemical impedance spectroscopy (EIS) tests, which were also conducted on the electrochemical workstation, an amplitude of 5 mV and frequency range of 10 MHz–100 kHz were applied. Self-discharge behaviors of the assembled ZHSs were studied by recording their open-circuit voltage over hold time, after the ZHSs were repeatedly charged/discharged for 5 cycles at 0.1 A g^−1^ and then kept at an expected voltage (e.g., 1.8 V) for 30 min through a constant voltage technique.

### Characterizations of Materials and Electrodes

Micro-morphologies of materials and electrodes were observed by scanning electron microscopy (SEM, Zeiss Supra 55VP, equipped with EDS analysis technique) and high-resolution transmission electron microscopy (TEM, Tecnai G2 F30). Phase composition was studied by X-ray diffraction (XRD) analyzer (Bruker D8 Discover Diffractometer). Surface pore structure, crystallinity and functional groups of the fibrous carbon materials were analyzed by a Brunauer–Emmett–Teller (BET) analyzer (ASAP 2020M+C), a laser Raman spectrometer (Gloucestershire, UK) with a Renishaw He–Ne laser source (17 mW at 633 nm) and X-ray photoelectron spectroscopy (XPS) technique (MDTC-EQM20-01), respectively. To reveal energy storage mechanism of the fibrous carbon cathodes, they were charged/discharged using a small current density of 0.1 A g^−1^ to different voltages in ZHS coin cells, and then taken out and washed with deionized water for 3 times to remove residual electrolyte. Their micro-morphologies and composite phases were studied.

## Results and Discussion

### Materials Characteristics

**Fig. 1:**
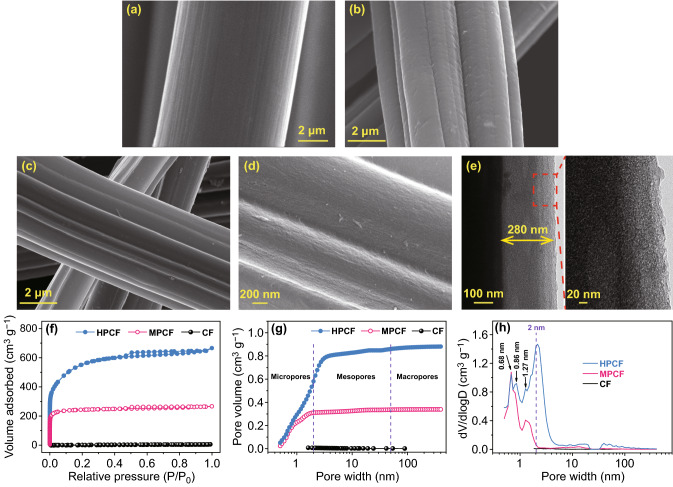
SEM images of **a** CF, **b** MPCF, and **c**, **d** HPCF samples. **e** TEM images of HPCF surface. **f** N_2_ adsorption/desorption isotherms, **g** pore volume integral curves, and **h** pore size distribution curves of the fibrous carbon samples

Figure 1 shows micro-morphology and pore structure of the fibrous carbon materials with different surface conditions. Carbon fibers (CFs) such as PAN-based commercial carbon fiber products are characterized by high strength, good electrical conductivity and good chemical stability, and thus have been widely used as substrates and current collectors for electrodes in electrochemical energy storage systems [39, 40]. However, CF has a relatively smooth surface with shallow grooves along length direction (Figs. 1a and S1). Its BET surface area and pore volume is only 10 m^2^ g^−1^ and 0.006 cm^3^ g^−1^, respectively (Fig. 1f–h and Table S1). Therefore, CF can hardly store charges through electric-double layer mechanism. For steam-activated carbon fiber (denoted as MPCF sample), there are deep grooves and many potholes on its surface (Figs. 1b and S1), and a high specific surface area of 810 m^2^ g^−1^ is achieved (Fig. 1f). Pore structure analysis in Fig. 1f–h and Table S1 illustrate that most of pores on MPCF surface are smaller than 2 nm, suggesting that MPCF has a micropore-dominated pore structure. After a further activation process of MPCF using KOH as activation agent, a hierarchically porous structure containing both micropores and mesopores/macropores forms inside HPCF sample. Volume of micropores, mesopores and macropores is 0.581, 0.279, and 0.021 cm^3^ g^−1^, respectively. Such a hierarchically porous carbon layer with thickness of about 280 nm can be observed on the fibrous carbon surface (Fig. 1c–h. The obtained sample is denoted as HPCF). Etching of carbon by KOH during the activation treatment leads to a slightly reduced fiber diameter from 7.4 μm in MPCF to 7.2 μm in HPCF. Besides, the activation treatment does not cause fracture of the fibrous carbon (Fig. S2). As a result, HPCF keeps a relatively high electrical conductivity of 3 S cm^−1^ (even though the value is inevitably lower than that of 21 S cm^−1^ for MPCF), which is beneficial for electron transport during electrochemical reactions. TEM images in Fig. 1e intuitively exhibit numerous pores (i.e., white dots in the right image) on HPCF surface. More specifically, different from micropore-dominated MPCF surface, many mesopores and a few macropores co-exist with abundant micropores on HPCF surface, i.e., HPCF surface has a hierarchically porous structure. As a result, specific surface area of HPCF is as high as ~ 2000 m^2^ g^−1^. Interestingly, micropores on HPCF surface and MPCF surface are very similar in size distribution (Fig. 1h). Although micropore volume is different for the two fibrous carbon materials, the micropores with size centered at 0.68, 0.86, and 1.27 nm are found from both of them. Highly porous surface and hydrophilic character make MPCF and HPCF easy to absorb moisture from air, as confirmed by thermogravimetric analysis in Fig. S3. Such a feature is beneficial for the two fibrous carbon materials to be effectively infiltrated by aqueous electrolytes in ZHSs, thus achieving good electrochemical performance.

**Fig. 2:**
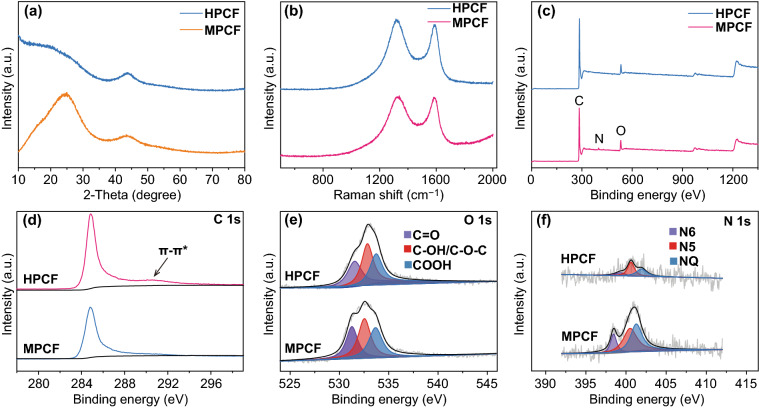
**a** XRD patterns, **b** Raman spectra, and **c-f** of XPS spectra of MPCF and HPCF samples. **c** is XPS full spectra and **d-f** are fine spectra of C 1s, O 1*s*, and N 1s

In the XRD pattern of MPCF (Fig. 2a), the diffraction peaks at 2*θ* = 26° and 44° are indexed to (002) and (101) plane, respectively, of graphitic carbon (JCPDS No. 75-1621). These diffraction peaks possess relatively weak intensity and broad width compared with that of CF sample (Fig. S4), suggesting a reduced order degree of graphitic structure of MPCF surface [41, 42], For HPCF sample, intensity of (002) peak notably decreases, and meanwhile, diffraction intensity significantly increases in the low-angle scatter. This phenomenon is caused by the presence of numerous pores on the surface of HPCF [41, 42], which is consistent with BET and TEM analysis in Fig. 1. D band and G band, representing disordered carbon structure and graphitic carbon structure, respectively, are observed from Raman spectra of MPCF and HPCF samples in Fig. 2b. Porous surface leads to a high intensity ratio (> 1.00) of D band to G band for both MPCF and HPCF samples, and relatively, HPCF has a higher intensity ratio, demonstrating that its surface contains more defect sites.

XPS technique is further applied to analyze surface functional groups of MPCF and HPCF samples. As displayed in Fig. 2c–f, signals of O and N heteroatoms are detected in XPS full spectra of MPCF and HPCF. Energy-dispersive spectroscopy (EDS) mapping images in Fig. S5 also confirm the uniform distribution of C, O, and N elements on HPCF surface. According to XPS analysis result, O and N content in MPCF sample is 8.4 and 2.1 at.%, respectively, while HPCF has a lower O content of 5.7 at.% and N content of 1.1 at.%. This is because during annealing process under N_2_ atmosphere, N cannot be effectively doped into carbon materials if other N sources such as melamine, ammonia and urea are not provided, and instead, O and N atoms release from carbon surface at high temperature, resulting in the decreased content of O and N heteroatoms during the activation process to prepare HPCF from MPCF [43, 44]. In C 1*s* fine spectra (Fig. 2d), we can find the *π*–π* satellite peak at ~ 291.0 eV for HPCF sample, which is considered to originate from delocalized electrons (e.g., aromatic rings) [45]. As confirmed above that there are numerous pores on HPCF surface, ultra-thin carbon shells like graphene may form between these pores and thus generate delocalized electrons. Besides, hydrophilic oxygen functional groups such as C–OH at 532.6 eV and COOH at 533.7 eV are detected on the surface of MPCF and HPCF (Fig. 2e), which can promote infiltration of active sites on the fibrous carbon surface by aqueous electrolytes [46]. In MPCF and HPCF samples, N heteroatom exists in the form of pyrrolic N (N5), pyridinic N (N6) and graphitic N (NQ), as analyzed in Fig. 2f. N doping is considered to have a positive impact on Zn^2+^ ion storage by carbon cathodes in ZHSs [22]. From this point, higher O/N content of carbon cathodes may be favorable for Zn^2+^ storage.

### Electrochemical Properties

**Fig. 3:**
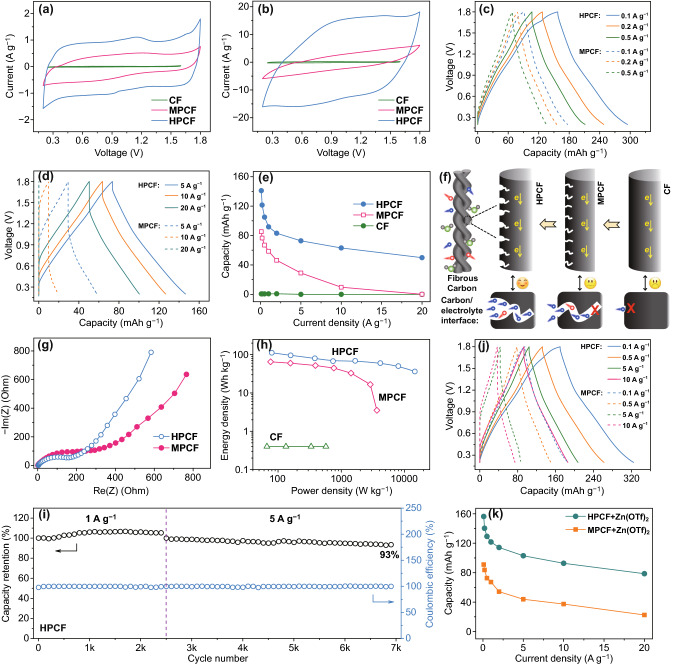
Electrochemical performance of the fibrous carbon cathode-based ZHSs with ZnSO_4_ aqueous electrolyte: CV curves at **a** 5 and **b** 100 mV s^−1^, GCD profiles at **c** 0.1–0.5 and **d** 5–20 A g^−1^, **e** discharge capacity values at various current densities, **f** schematic of energy storage on the fibrous carbon cathodes with different surface conditions, **g** EIS spectra, **h** Ragone plots, and **i** cycling behavior. **j** GCD profiles and **k** capacity summary of the fibrous carbon cathode-based ZHSs with Zn(OTf)_2_ aqueous electrolyte

Electrochemical behaviors of the fibrous carbon materials as free-standing cathodes for ZHSs with 2 M ZnSO_4_ aqueous electrolyte are displayed in Fig. 3a–i. As expected, non-porous surface decides poor electrochemical activity of CF cathode in ZHSs, reflecting by negligible response current on CV curve in Fig. 3a and very small capacity determined by GCD tests in Fig. S6. On the contrary, MPCF and HPCF cathodes with highly porous surface possess high electrochemical activity in ZHS systems, accompanying with large response current in voltage window of 0.2–1.8 V versus Zn^2+^/Zn. Working potentials of HPCF electrode and zinc electrode were also determined in a three-electrode system (Fig. S7). To be specific, the CV curves of MPCF and HPCF cathode-based ZHSs can be regarded as a combination of a rectangle and redox peaks, which are significantly different from rectangle-shaped CV curves of carbon-based symmetric supercapacitors (Fig. S8), because ZHS systems contain both battery-like reaction on zinc anodes and electric double-layer capacitive behavior on carbon cathodes. It is worth emphasizing that compared with aqueous symmetric supercapacitors, the use of zinc anodes with relatively low redox potential (− 0.76 V vs. SHE) is beneficial for ZHSs to achieve higher operating voltage and thus high energy density. Furthermore, as scan rate increases to a large value such as 100 mV s^**−1**^ (Fig. 3b), CV curves of the fibrous carbon cathodes show varying degrees of distortion. By comparison, distortion in CV curves of MPCF cathode is much more serious than that of HPCF cathode, suggesting that HPCF cathode has faster kinetics of electrochemical reactions than MPCF.

Capacity and rate performance of the fibrous carbon cathodes were determined by GCD measurements. Inert surface of CF cathode leads to a very small specific capacity of 0.6 mAh/g at a charge/discharge current density of 0.1 A g^−1^ (Fig. S6). For both MPCF and HPCF cathodes, their highly porous surface provides abundant active sites for ion adsorption/desorption, thus bringing a 100-fold increase in capacity (Fig. 3c–e). HPCF cathode shows a much higher discharge capacity than MPCF cathode (141 vs. 85 mAh g^−1^ at 0.1 A g^−1^). There is no doubt that HPCF cathode’s higher capacity is associated with its higher specific surface area (2000 vs. 810 m^2^ g^−1^ for MPCF). But it is also undeniable that at large charge/discharge current densities such as 5–20 A g^−1^, specific capacity of MPCF cathode decreases dramatically, while that of HPCF cathode keeps at a relatively high level, demonstrating a better rate capability of HPCF cathode (Fig. 3d, e). That is, specific capacity of the fibrous carbon cathodes is not simply proportional to their specific surface area. According to previous researches about porous carbon materials for supercapacitors [23, 47], micropores are beneficial for providing electrochemical active sites for ion adsorption/desorption, thus resulting in a high capacity, while mesopores and macropores generally serve as electrolyte reservoir to shorten ion transport distance, thus optimizing rate performance. As we have discussed above, MPCF surface is dominated by micropores, while HPCF has a hierarchically porous surface. Although a relatively high capacity is achieved at slow charge/discharge rates for MPCF cathode due to abundant micropores on its surface, the absence of mesopores and macropores leads to a poor rate performance. In contrast, HPCF surface contains not only numerous micropores to bring a high capacity, but also mesopores and macropores to guarantee a superior rate capability. Besides, continuous fibrous carbon can transport electrons rapidly, which also contributes to the superior rate capability of HPCF cathode. Such relationship between surface pore structure and electrochemical performance of fibrous carbon cathodes is schematically illustrated in Fig. 3f. EIS spectra at open-circuit voltage in Fig. 3g and their fitting result in Fig. S9 provide more evidence. Smaller semicircle on the EIS spectrum of HPCF cathode proves that HPCF has a smaller charge-transfer resistance at electrode/electrolyte interface and fast kinetics during electrochemical reactions, in comparison with MPCF cathode [48]. Meanwhile, intercept of these EIS spectra at high-frequency zone on the horizontal axis is very small, indirectly reflecting high electric conductivity of the two fibrous carbon cathodes. As expected, HPCF cathode-based ZHS is capable to deliver a high energy density of 112 Wh kg^−1^ and a high power density of 14.5 kW kg^−1^ (Fig. 3h). The performance is significantly superior to that of CF and MPCF cathode-based ZHSs and many previously reported ZHSs (as summarized in Table S2). Furthermore, HPCF cathode-based ZHS shows good long-term cycling stability. As shown in Fig. 3i, discharge capacity of HPCF cathode does not degrade after 2500 charge/discharge cycles at 1 A g^−1^, and further 4,500 cycles at 5 A g^−1^ only causes a 7% capacity loss. Meanwhile, during the long-term cycle test, Coulombic efficiency always keeps around 100%, showing good reversibility of electrochemical reactions on HPCF cathode. Even at a large current of 20 A g^−1^, the capacity of HPCF cathode keeps 93% over 6000 charge/discharge cycles (Fig. S10). This suggests that the hierarchically porous structure on HPCF surface is stable enough to bear large current impact.

Electrochemical behaviors of the fibrous carbon cathodes are also studied in 2 M Zn(OTf)_2_ aqueous electrolyte. As displayed in Figs. 3j, k and S11, both MPCF and HPCF cathodes can repeatedly charge/discharge in a voltage window of 0.2–1.8 V versus Zn^2+^/Zn. The difference between them is that HPCF cathode shows much higher capacity at various charge/discharge current densities than MPCF cathode. For instance, discharge capacity of HPCF and MPCF cathode is 156 and 91 mAh g^−1^, respectively, at a low current density of 0.1 A g^−1^, and the value becomes 79 mAh g^−1^ for HPCF cathode and 23 mAh g^−1^ for MPCF cathode at a large current density of 20 A g^−1^. As a result, maximum energy density of HPCF cathode-based ZHS reaches 127 Wh kg^−1^ and maximum power density reaches 15.3 kW kg^−1^, much higher than those values of MPCF cathode-based ZHS (Fig. S12). In a word, HPCF cathode is superior to MPCF cathode in capacity, rate performance and energy density. This is similar to the phenomenon observed in ZnSO_4_ aqueous electrolyte, and once again confirms the superiority of the hierarchically porous surface of HPCF. Besides, compared with ZnSO_4_ electrolyte, utilization of Zn(OTf)_2_ electrolyte endows MPCF and HPCF cathodes with better rate performance and enhanced capacity. An important reason is that a smaller charge-transfer resistance at electrode/electrolyte interface, i.e., faster kinetics of electrochemical reactions, can be realized in Zn(OTf)_2_ electrolyte, as revealed by EIS analysis in Fig. S13. More deeply, Zn(OTf)_2_ aqueous electrolyte has higher ionic conductivity and better Zn^2+^ mobility due to weak restricting effect by anions, in comparison with ZnSO_4_ aqueous electrolyte [49]. Despite these, Zn(OTf)_2_ is much expensive than ZnSO_4_ (61.9 vs*.* 0.24 ¥ g^−1^), but maximum energy density and maximum power density of HPCF cathode in the two aqueous electrolytes do not show significant difference (Fig. S12). Therefore, HPCF cathode-based ZHS with ZnSO_4_ electrolyte may be more attractive from application point of view. Analysis below is mainly centered on the ZHSs with ZnSO_4_ aqueous electrolyte.

### Mechanism Investigation

**Fig. 4:**
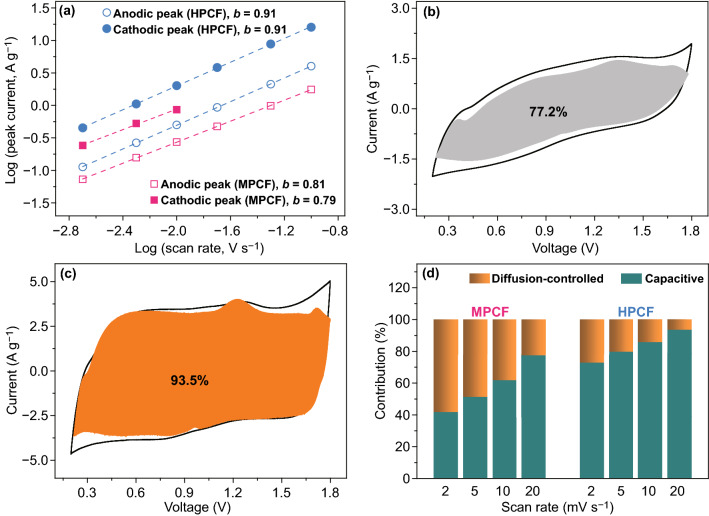
Electrochemical kinetics analysis: **a** relationship between peak current and scan rate; total current at 20 mV s^−1^ (black line) and capacitive process-contributed current (shadow area) of **b** MPCF and **c** HPCF cathode; **d** contribution ratios of capacitive process and diffusion-controlled process to cathode capacity

Electrochemical kinetics are investigated to reveal the effects of surface conditions on electrochemical behaviors of the fibrous carbon cathodes. For anodic peak at ~ 1.2 V and cathodic peak at ~ 0.9 V on the CV curves of MPCF and HPCF cathode-based ZHSs, their peak current (*i*) can be expressed as a function of scan rate (*v*) through Eq. (1) [18, 50]:

1 $$i = av^{b}$$

where *a* and *b* are variable parameters. For a capacitive process, its *b* value is 1, and for a diffusion-controlled process, its *b* value is 0.5. As summarized in Fig. 4, the anodic peak and the cathodic peak of HPCF cathode correspond to a same *b* value of 0.91, and those of MPCF cathode correspond to *b* values of 0.79 and 0.81 (note that cathodic peaks of MPCF cathode become blurred at large scan rates). This proves that fast capacitive process dominates electrochemical energy storage inside HPCF cathode-based ZHS, but electrochemical reactions inside MPCF cathode-based ZHS are notably affected by diffusion-controlled process. For an electrochemical reaction, its total response current (*i*) at a scan rate of *v* can be expressed by Eq. (2) [50, 51]:

2 $$i = k_{1} v + k_{2} v^{1/2}$$

in which *k*_1_*v* and *k*_2_*v*^1/2^ represent the current contributed by capacitive process and diffusion-controlled process, respectively. The fitting results are shown in Fig. 4b–d. For MPCF cathode, 41.8–77.2% of its capacity originates from capacitive process, while for HPCF cathode, the value is 72.8–93.5%. Since capacitive process actually stands for fast kinetics, the higher contribution by capacitive process well explains HPCF cathode’s superior rate capability. In addition, diffusion-controlled capacity for both MPCF and HPCF cathodes may be associated with N/O heteroatom functional groups on the surface of the two fibrous carbon cathodes, considering that N/O heteroatoms can interact with electrolyte ions through pseudocapacitive behaviors [34]. Furthermore, we would like to mention that at low scan rates (equal to low charge/discharge current densities), diffusion-controlled process accompanying with relatively low electrochemical kinetics has a notable effect on the specific capacity of the carbon cathodes. As a result, the specific capacity decreases rapidly as current density increases from 0.1 to 2 A g^−1^ (Fig. 3e).

**Fig. 5:**
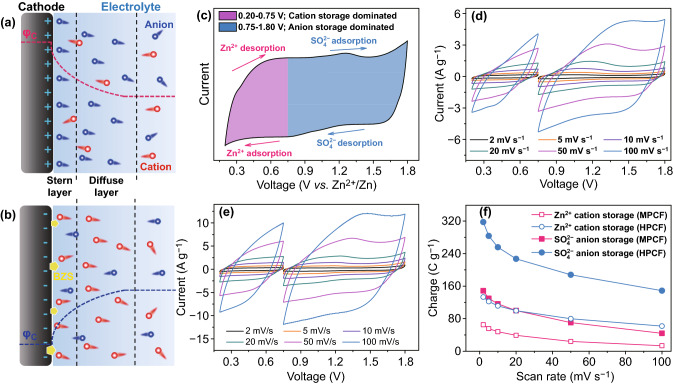
Schematics of ion storage by carbon cathodes: **a** anion storage dominated process, **b** cation storage dominated process, and **c** ion storage in different voltage ranges of ZHSs. CV curves in voltage windows of 0.2–0.75 and 0.75–1.80 V for **d** MPCF and **e** HPCF cathode-based ZHSs with 2 M ZnSO_4_ aqueous electrolyte. **f** Charge amount stored on MPCF and HPCF cathodes at different scan rates

As have pointed out in our previous work and some other literature, electrochemical energy storage of carbon cathode-based ZHSs is realized by anion storage at high voltage and cation storage at low voltage [9, 14, 52] According to Nernst equation, redox potential of Zn^2+^/Zn (*φ*_A_) in ZnSO_4_ aqueous electrolyte at 298 K can be calculated using Eq. (3):

3 $$\phi_{A} = - 0.76 + 0.01284 \times \ln (c)$$

in which *c* is molar concentration of the used ZnSO_4_ aqueous electrolyte. For instance, redox potential of Zn^2+^/Zn is − 0.75 V (vs*.* SHE) in 2 M ZnSO_4_ aqueous electrolyte. Actually, even when *c* varies in a large range of 0.1–3 M, fluctuation of *φ*_A_ is less than 0.04 V. Therefore, even though charge/discharge processes can cause polarization and fluctuation of Zn^2+^ concentration around zinc anodes, it is acceptable to assume that potential of zinc anodes is always − 0.75 V (vs*.* SHE) in ZHS systems. Such an assumption is also supported by experimental result in Fig. S14 and previous literature [52]. Therefore, when MPCF and HPCF cathode-based ZHSs are charged/discharged in voltage range of 0.75–1.80 V, cathode potential (denoted as *φ*_C_) is above 0 V versus SHE, which should be generated by positively charged electrode and negatively charged electrolyte at the electrode/electrolyte interface, as illustrated in Fig. 5a. While when the ZHSs are charged/discharged in voltage range of 0.20–0.75 V, cathode potential is below 0 V versus SHE, which should be generated by negatively charged electrode and positively charged electrolyte at the electrode/electrolyte interface, as illustrated in Fig. 5b. Based on the above discussion, main reactions of MPCF and HPCF cathodes in ZnSO_4_ aqueous electrolyte can be divided into two parts, i.e., Zn^2+^ cation adsorption/desorption in voltage rang of 0.20–0.75 V and SO_4_^2−^ anion adsorption/desorption in voltage rang of 0.75–1.80 V. More specifically, as depicted in Fig. 5c, when the ZHSs are discharged from 1.80 to 0.75 V, SO_4_^2−^ anions gradually desorb from the cathode surface, and further discharge process below 0.75 V corresponds to Zn^2+^ cation adsorption on the cathode surface. Conversely, when the ZHSs are charged from 0.2 to 0.75 V, Zn^2+^ cations gradually desorb from the cathode surface, and further charge process above 0.75 V corresponds to SO_4_^2−^ anion adsorption on the cathode surface.

For the above reason, the MPCF and HPCF cathodes-based ZHSs were separately scanned using CV technique in the voltage windows of 0.20–0.75 and 0.75–1.80 V (Fig. 5d, e). Charge amount stored by the cathode in these two voltage windows was calculated (Fig. 5f). Note that HPCF cathode can store more Zn^2+^ cations and SO_4_^2−^ anions than MPCF cathode, and in particular, electrochemical storage of Zn^2+^ cations and SO_4_^2−^ anions on HPCF cathode is significantly superior to that on MPCF cathode at high scan rates. These are consistent with higher capacity and better rate capability of HPCF cathode. Besides, for both MPCF and HPCF cathodes, a high proportion of their stored charge originates from SO_4_^2−^ anion storage, while Zn^2+^ cation storage accounts for a low proportion. For instance, 318 C g^−1^ SO_4_^2−^ anions and 149 C g^−1^ Zn^2+^ cations are stored by HPCF cathode at 2 mV s^−1^.

**Fig. 6:**
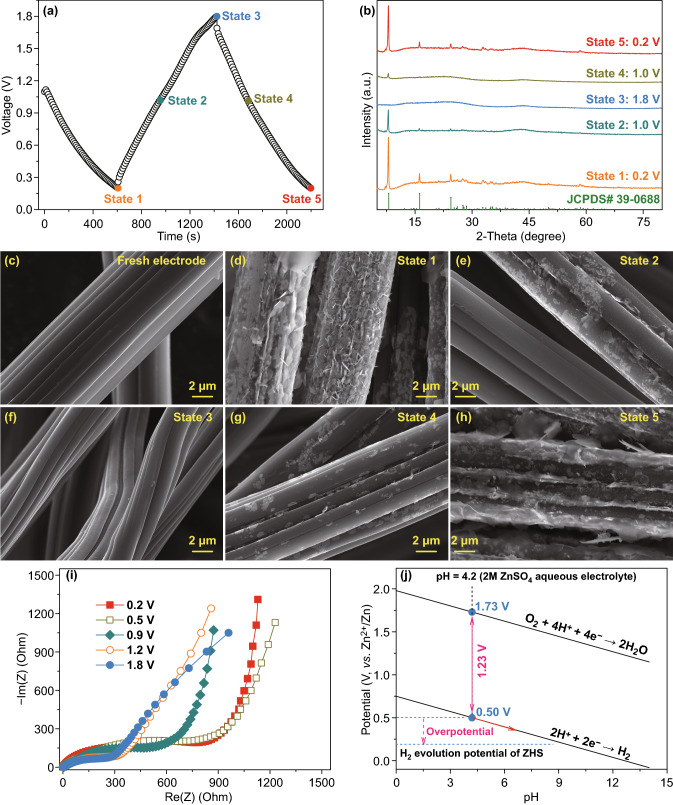
**a** HPCF cathode was charged/discharged to different states; **b** XRD patterns, **c**–**h** SEM images and **i** EIS spectra of HPCF cathode in ZHSs at different charge/discharge states. **j** Potential of hydrogen/oxygen evolution in aqueous systems

We further investigated energy storage mechanism of HPCF cathode in ZHSs by tracking evolution of its phase composition and micro-morphology during charge/discharge processes. Most of currently reported carbon cathodes of ZHSs are composed of active materials (i.e., synthesized carbon materials), conductive additives, binder and current collectors, but the latter three compounds affect formation and identification of charge/discharge products. The free-standing characteristic of HPCF cathode makes it easy to precisely identify charge/discharge products generated on the cathode. As displayed in Figs. 6a–h and S15, when HPCF cathode is firstly discharged from original state to 0.2 V (i.e., state 1 in Fig. 6d), many flakes appear on HPCF surface. Combined with XRD analysis in Fig. 6b, these flakes are identified as basic zinc sulfate (JCPDS No. 39-0688), with chemical formula of Zn_4_SO_4_(OH)_6_·5H_2_O or ZnSO_4_·3Zn(OH)_2_·5H_2_O (BZS). In subsequent charging process, the BZS flakes gradually disappear: only a few BZS flakes remain in the cathode at 1.0 V (i.e., state 2 in Fig. 6e), and no BZS can be detected in the fully charged cathode (i.e., state 3 in Fig. 6f). Further discharging induces the re-generation of BZS flakes (states 4 and 5 in Fig. 6g, h), and especially when HPCF cathode is discharged from 1.0 to 0.2 V, amount of the BZS flakes dramatically increases. As pointed out in previous literature, BZS flakes form through Eq. (4) [14, 53]:

4 $$4{\text{Zn}}^{2 + } + 6{\text{OH}}^{ - } + {\text{SO}}_{4}^{2 - } + 5{\text{H}}_{2} {\text{O}} \leftrightarrow {\text{Zn}}_{4} {\text{SO}}_{4} ({\text{OH}})_{6} \cdot 5{\text{H}}_{2} {\text{O}} \downarrow$$

Considering that solubility product constant (*k*_sp_) of BZS precipitation is expressed as Eq. (5):

5 $$\ln (k_{{{\text{sp}}}} ) = 4\ln \left[ {c_{{({\text{Zn}}^{2 + } )}} } \right] + 6\ln \left[ {c_{{({\text{OH}}^{ - } )}} } \right] + \ln \left[ {c_{{({\text{SO}}_{4}^{2 - } )}} c_{{({\text{SO}}_{4}^{2 - } )}} } \right]$$

in which *c*_(x)_ is molar concentration of X (X represents Zn^2+^, OH^−^ and SO_4_^2−^). Obviously, concentration of Zn^2+^ cations and OH^−^ anions is more influential in the generation of BZS precipitation, in comparison with that of SO_4_^2−^ anions. As have discussed in Fig. 5, when HPCF cathode is gradually discharged to 0.2 V, Zn^2+^ cations aggregate around HPCF surface, accompanying with increased Zn^2+^ concentration. At the same time, due to slightly acidic characteristic of ZnSO_4_ aqueous electrolyte, H^+^ adsorption on HPCF surface is inevitable during discharge process, which will cause increased pH value of the electrolyte (being equivalent to increased OH^−^ concentration). Then, flake-like BZS precipitation appears on HPCF surface, as depicted by Eqs. (4) and (5), and its amount increases with decreasing discharge voltage of HPCF cathode-based ZHS. While during charging process, Zn^2+^ and H^+^ release from HPCF cathode and BZS precipitation tends to dissolve into electrolyte. It should be emphasized that the BZS has multiple influences on electrochemical properties of HPCF cathode-based ZHSs. On the one hand, insulating BZS flakes cover on HPCF surface, resulting in increased charge-transfer resistance during electrochemical reactions (Fig. 6i). In this view, generation of BZS is unfavorable to achieving a superior rate performance for HPCF cathode. This is consistent with the fact that Zn^2+^ storage at low voltage has poor kinetics, as discussed in Fig. 5d–f. On the other hand, BZS expands voltage window and thus enhance energy density of HPCF cathode-based ZHSs. For pure 2 M ZnSO_4_ aqueous electrolyte whose pH is about 4.2, the potential of hydrogen evolution and oxygen evolution is calculated to be 0.50 and 1.73 V (vs. Zn^2+^/Zn) in theory (Fig. 6j). But working voltage window of HPCF cathode is 0.20–1.80 V (vs. Zn^2+^/Zn), suggesting that hydrogen evolution reaction is notably suppressed in ZHSs. As proved by Qin et al*.*, BZS can bring large overpotential for hydrogen evolution [54]. Besides, the increased pH value of the electrolyte when HPCF cathode-based ZHS is gradually discharged to 0.2 V will also lower hydrogen evolution potential, as illustrated by the red arrow in Fig. 6j. These two factors effectively inhibit hydrogen evolution reaction and expand voltage window of the aqueous ZHSs. Moreover, no other charge/discharge products are detected on HPCF cathode except BZS (Fig. 6b–h), indirectly confirming the energy storage mechanism of ion adsorption/desorption of HPCF cathode.

### Self-discharge Behaviors

Self-discharge behavior has crucial effect on practical application of electrochemical energy storage systems including ZHSs, because serious self-discharge means considerable loss of energy for electrochemical devices during their storage. To assess self-discharge performance of MPCF and HPCF cathode-based ZHSs, their open-circuit voltage was continuously recorded after they were charged to an expected voltage (e.g., 1.8 V. Detailed procedures are illustrated in Fig. S16). For comparison, self-discharge behaviors of some other representative electrochemical energy storage systems using 2 M ZnSO_4_ aqueous electrolyte were also tested, including an AC//AC symmetric supercapacitor, AC//Zn ZHS and V_10_O_24_·12H_2_O//Zn Zn-ion battery, and their basic information such as specific capacity are provided in Fig. S17.

**Fig. 7:**
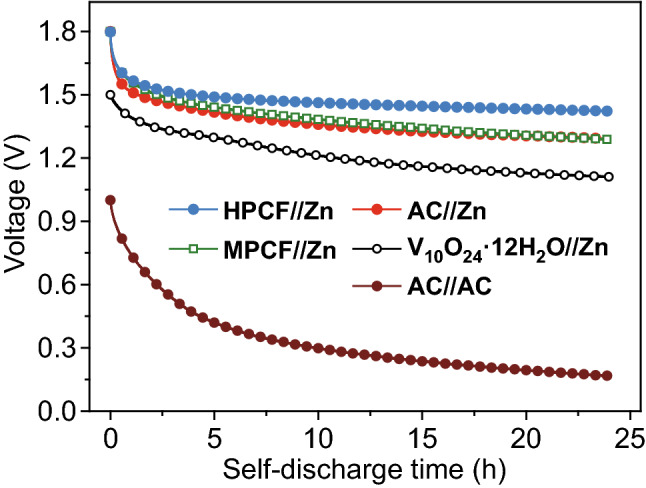
Self-discharge behaviors of MPCF and HPCF cathode-based ZHSs and some other electrochemical energy storage systems in 2 M ZnSO_4_ aqueous electrolyte

Figure 7 exhibits that open-circuit voltage of AC//AC symmetric supercapacitor decreases rapidly and retains only 17% of initial value after 24 h hole time, showing a very serious self-discharge behavior. Such serious self-discharge behavior is commonly seen in carbon-based symmetric supercapacitors [32, 55]. In sharp contrast, AC//Zn ZHS system exhibits much better anti-self-discharge feature than AC//AC symmetric supercapacitor, which indicates that utilization of zinc anode is beneficial for optimizing anti-self-discharge performance. This is because low and stable zinc anode potential (− 0.75 V vs. SHE in 2 M ZnSO_4_ aqueous electrolyte) is helpful to stabilize electric field inside ZHSs, thereby restraining anion desorption from cathode surface and self-discharge of ZHS systems (as illustrated in Fig. S18). Consequently, other ZHSs and Zn-ion battery systems constructed with zinc anodes in Fig. 7 are also endued with slow self-discharge rate. Besides, HPCF cathode-based ZHS exhibits the optimal anti-self-discharge performance. Although its open-circuit voltage notably drops from initial voltage of 1.80 to 1.52 V in the first 2 h, the voltage then declines very slow, and a high voltage of 1.42 V is remained after 24 h hold time. In addition, when HPCF cathode-based ZHS is charged to an initial voltage of 1.4 and 1.6 V and then holds for 24 h at the condition of open circuit, 95% and 86% voltage retention is observed, respectively (Fig. S19), confirming the superior anti-self-discharge capability of HPCF cathode-based ZHS. MPCF cathode-based ZHS, by contrast, shows inferior anti-self-discharge performance. Since transport of electrolyte ions is difficult in carbon pores with small diameter and long length, carbon electrodes with such pore structure tends to suffer serious self-discharge due to charge redistribution after charging [35, 56]. From this point of view, ion transport is relatively easy inside hierarchical pores on HPCF surface, thereby leading to a better anti-self-discharge performance. Besides, the smaller amount of oxygen functional groups on HPCF surface also contributes to the better anti-self-discharge performance of HPCF cathode-based ZHS, because oxygen functional groups generally weaken static electrical force between carbon electrode and electrolyte ions [36, 55]. Overall, hierarchical pore structure and suitable oxygen functional groups of HPCF cathode and its coupling with zinc anodes contribute to good anti-self-discharge performance of the ZHSs.

## Conclusions

A surface engineering strategy was applied to design hierarchically porous structure on fibrous carbon surface with O/N heteroatom functional groups, and thus high-energy and anti-self-discharge ZHSs were realized. The fabricated fibrous carbon showed a high specific surface area, along with excellent electrochemical performance, such as high capacity, superior rate capability and exceptional cycling stability when used as free-standing cathodes of ZHSs. We demonstrated that the hierarchically porous surface of the fibrous carbon cathodes provided not only abundant active sites for divalent ion storage to achieve high capacity, but also optimized ion transport kinetics to realize superior rate performance. Meanwhile, hierarchical pore structure and suitable surface functional groups of the cathodes endowed ZHSs with high energy/power density and good anti-self-discharge performance. Mechanism investigation revealed that charge–discharge processes of the fibrous carbon cathodes involved cation adsorption/desorption and BZS formation/dissolution at low voltage and anion adsorption/desorption at high voltage. Although BZS led to low electrochemical kinetics of Zn^2+^ storage, it expanded the working voltage window of ZHSs. This work is believed to promote the development of high-performance cathode materials and electrochemistry theory of ZHS systems.

## Supplementary Information

Supplementary file1 (PDF 1269 kb)
