# Supplementary material for: Towards High-Energy and Anti-Self-Discharge Zn-Ion Hybrid Supercapacitors with New Understanding of the Electrochemistry
Source: Nanomicro Lett. 2021 Mar 18;13:95. doi: 10.1007/s40820-021-00625-3 (PMC8006207; doi:10.1007/s40820-021-00625-3)
Supplement: Supplementary file 1 — Supplementary file1 (PDF 1269 kb) [file 40820_2021_625_MOESM1_ESM.pdf]

Supporting Information for

Towards High-Energy and Anti-Self-Discharge Zn-Ion Hybrid Supercapacitors with New Understanding of the Electrochemistry

Yang Li [1], Wang Yang [2], Wu Yang [2], Ziqi Wang [1,] *, Jianhua Rong [1], Guoxiu Wang [2,] *, Chengjun Xu [3], Feiyu Kang [3], Liubing Dong [1,] *

[1] College of Chemistry and Materials Science, Jinan University, Guangzhou 511443, P. R. China

[2] Centre for Clean Energy Technology, University of Technology Sydney, NSW 2007, Australia

[3] Tsinghua Shenzhen International Graduate School, Tsinghua University, Shenzhen 518055, P. R. China

*Corresponding authors. E-mail: wangzq@jnu.edu.cn (Z. Wang);

guoxiu.wang@uts.edu.au (G. Wang); donglb@jnu.edu.cn (L. Dong)

S1 Experimental Section

Specific capacity (C, mAh g^{-1}), energy density (E, Wh kg^{-1}) and power density (P, W kg^{-1}) were determined by GCD tests, and their calculation formulas are as follows.

$$C = \frac{1}{3.6} \times I \times t \quad (S1)$$

$$E = \int U dC = \frac{I}{3.6} \times \int U dt \quad (S2)$$

$$P = 3600 \times \frac{E}{t} \quad (S3)$$

in which I is current density (unit: A g^{-1}), t is charge or discharge time (unit: s) and U (unit: V) is voltage during a charge or discharge process. Note that, since zinc foils serve as active materials and current collectors at the same time in ZHSs, their mass is excessive. Therefore, capacity, energy density and power density of the ZHSs were calculated based on the weight of cathode materials.

S2 Supplementary Figures and Tables

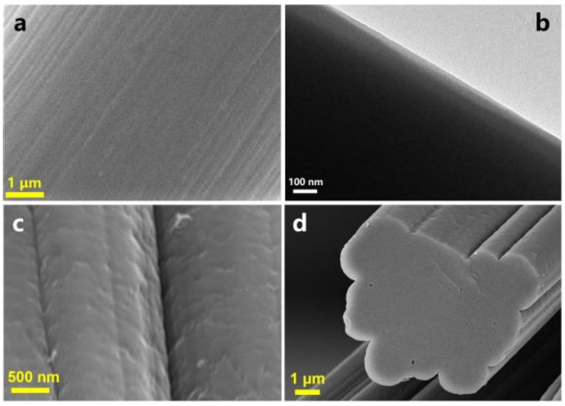

Fig. S1 (**a**) SEM image and (**b**) TEM image of CF; (**c**) (**d**) SEM images of MPCF. CF has a relatively smooth surface without porous carbon layer like that in HPCF (Fig. 1e). Cross-sectional SEM image of MPCF in (**d**) clearly shows that there are many deep grooves on the surface of the MPCF sample [S1].

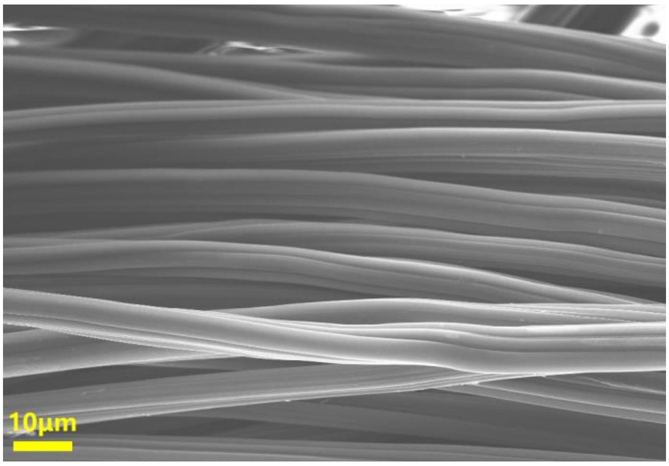

Fig. S2 SEM image at low magnification of HPCF. Broken fibers are hardly observed in HPCF sample.

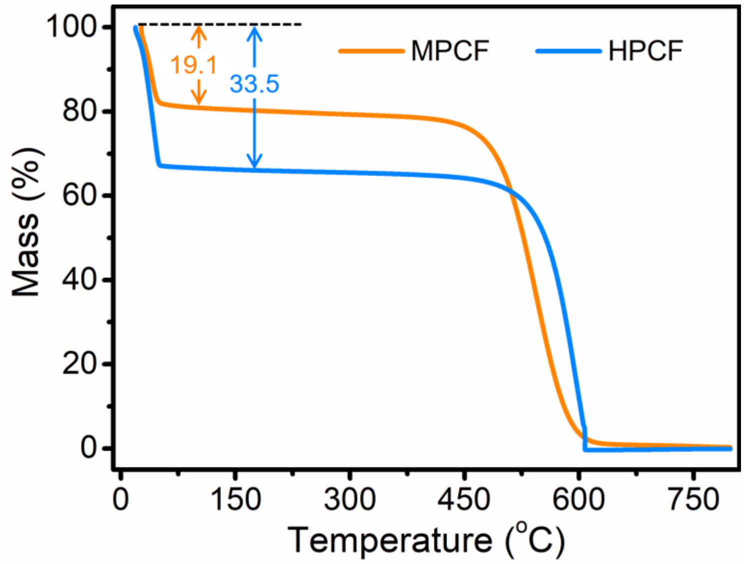

Fig. S3 Thermogravimetric curves of MPCF and HPCF. Absorbed water in MPCF and HPCF accounts for 19.1 and 33.5%, respectively.

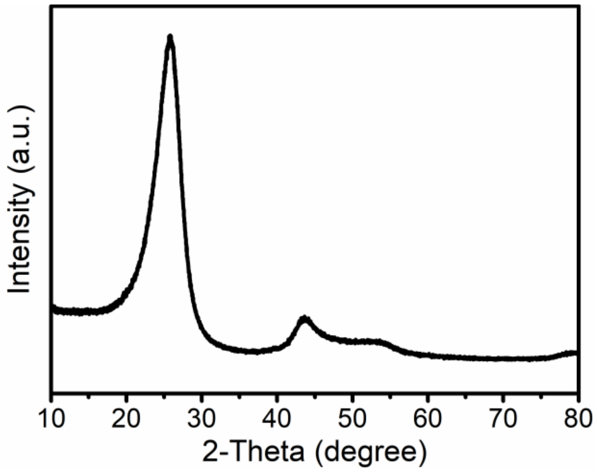

Fig. S4 XRD pattern of CF sample. The strong diffraction peaks at $2\theta=26^{o}$ and 44° suggest a high order degree of graphitic structure, *i.e.*, few pores on CF surface.

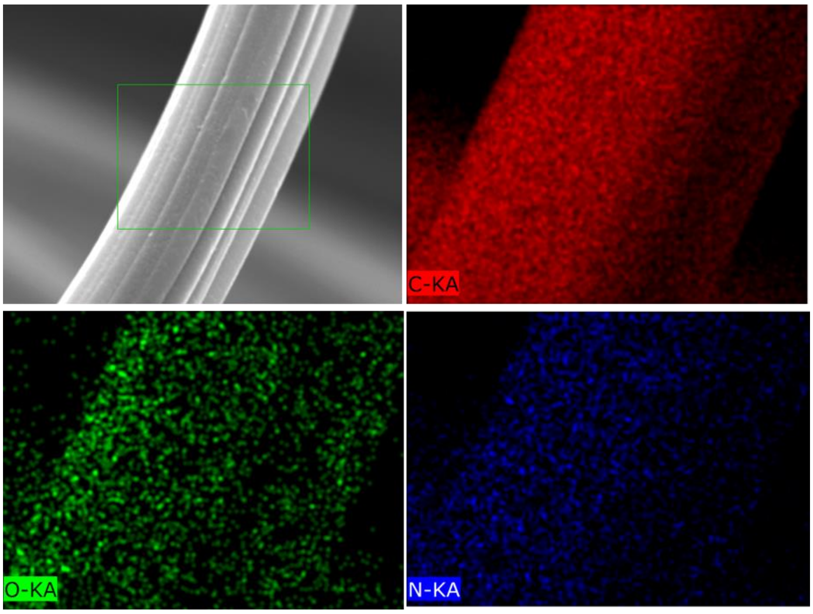

Fig. S5 EDS mapping images of C, O and N elements on HPCF surface

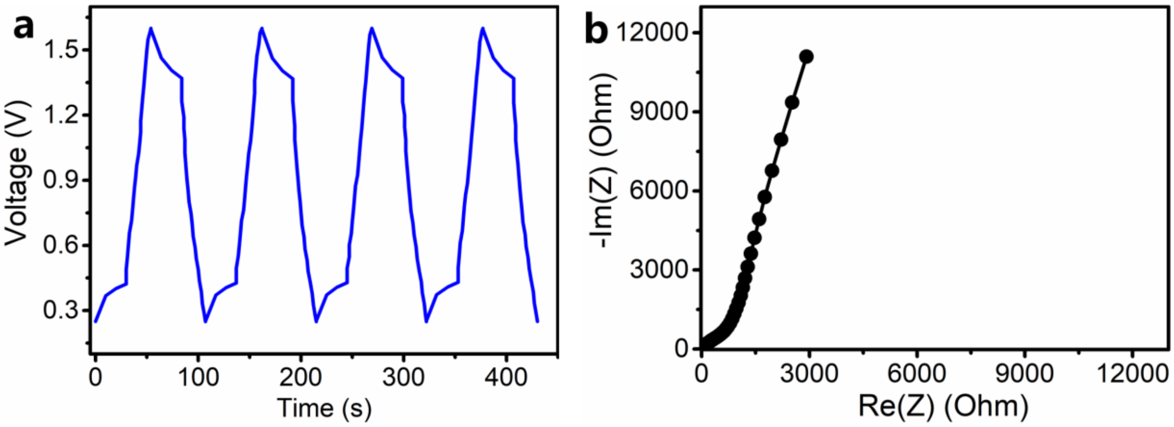

Fig. S6 (**a**) GCD curves at 0.1 A g^{-1} and (**b**) EIS spectrum of CF cathode-based ZHSs. Capacity of CF cathode is only ~0.6 mAh g^{-1}, which means that it almost does not possess electrochemical activity in ZHSs.

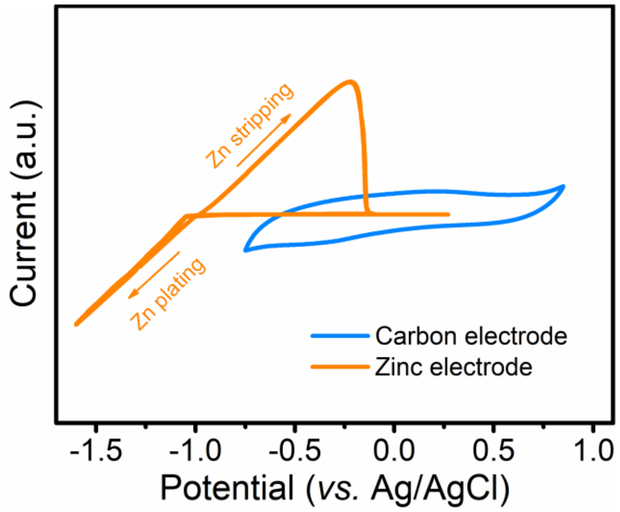

Fig. S7 CV curves at 2 mV s^{-1} of HPCF carbon electrodes and zinc electrodes in three-electrode systems, in which HPCF or metallic zinc serves as working electrode, stainless steel foil serves as counter electrode and Ag/AgCl electrode serves as reference electrode.

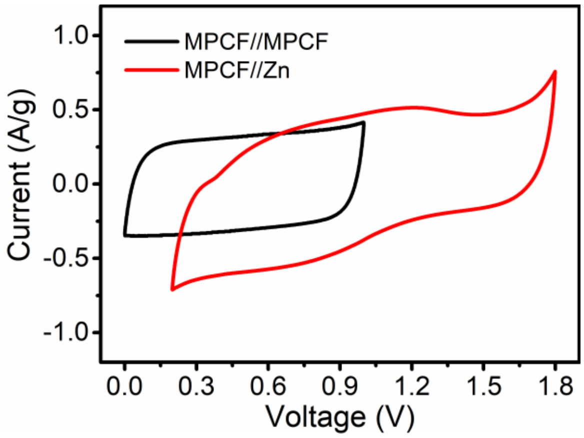

Fig. S8 CV curves at 5 mV s^{-1} of MPCF//MPCF symmetric supercapacitors and MPCF//Zn ZHSs. Both of the two systems use 2 M $ZnSO_4$ aqueous solution as electrolyte.

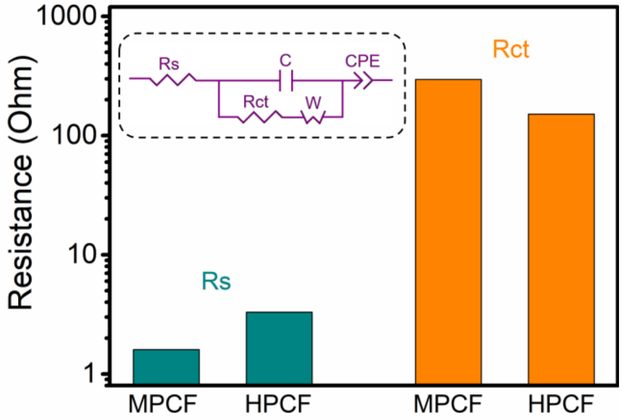

Fig. S9 Fitting data of EIS spectra of MPCF and HPCF cathodes in Fig. 3g. Inset is corresponding equivalent circuit model. R_s contains electrolyte resistance and contact resistance between electrodes and current collectors, and R_{ct} is charge-transfer resistance at electrode/electrolyte interface. R_s of MPCF and HPCF cathodes is very small (1.6 and 3.3 Ω), resulting from good electrical conductivity of these fibrous carbon cathodes. R_{ct} of MPCF and HPCF cathodes is 295.7 and 151.8 Ω, respectively. The much smaller R_{ct} value of HPCF cathode demonstrates its fast kinetics during electrochemical reactions.

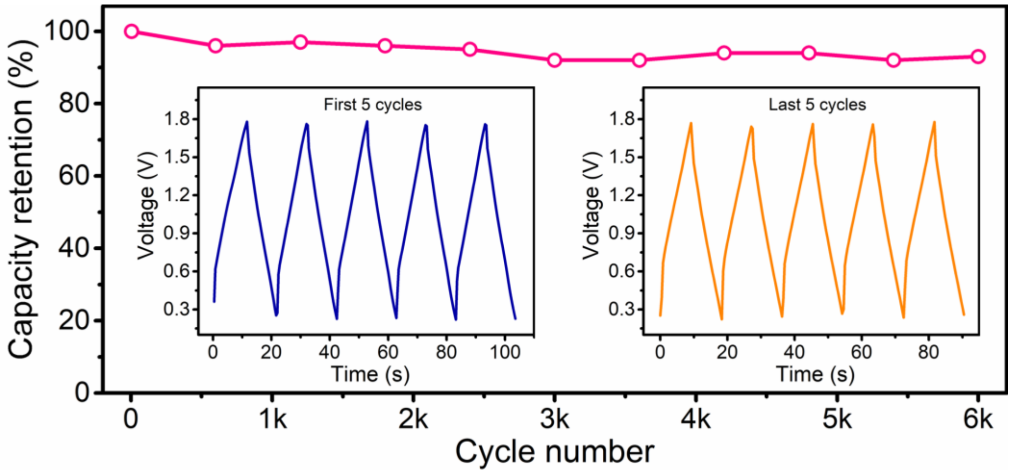

Fig. S10 Cycling behavior at 20 A/g of HPCF cathode-based ZHS with 2 M $ZnSO_4$ aqueous electrolyte. Capacity retention is 93% over 6000 charge/discharge cycles. Insets are the first and last 5 cycles of GCD curves.

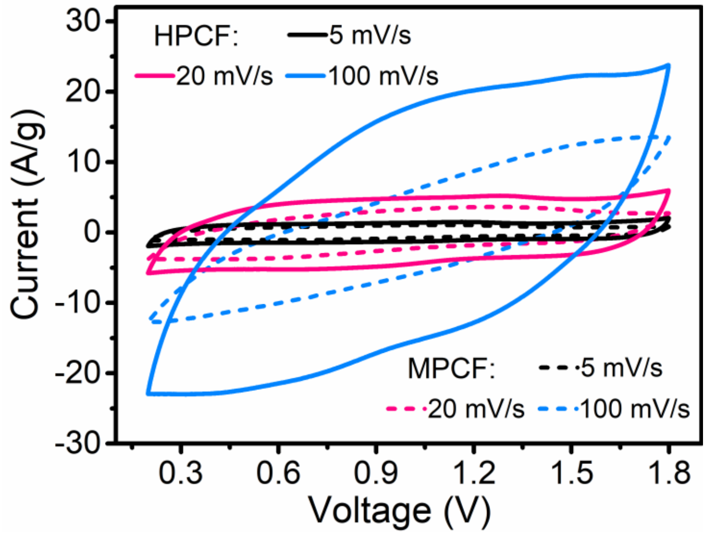

Fig. S11 CV curves of the fibrous carbon cathode-based ZHSs with 2 M $Zn(OTf)_2$ aqueous electrolyte

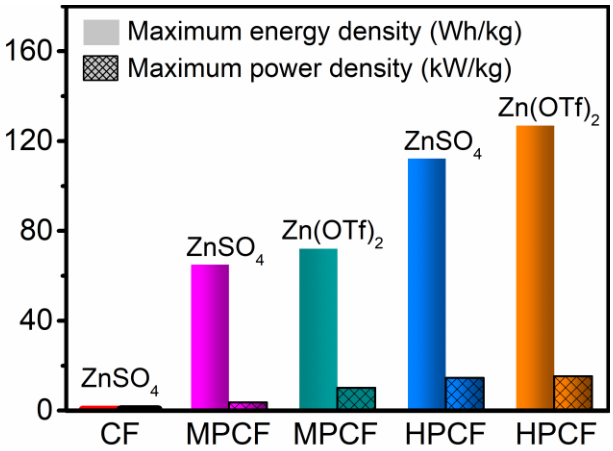

Fig. S12 Maximum energy density and maximum power density of MPCF and HPCF cathode-based ZHSs with 2 M $ZnSO_4$ and 2 M $Zn(OTf)_2$ aqueous electrolytes

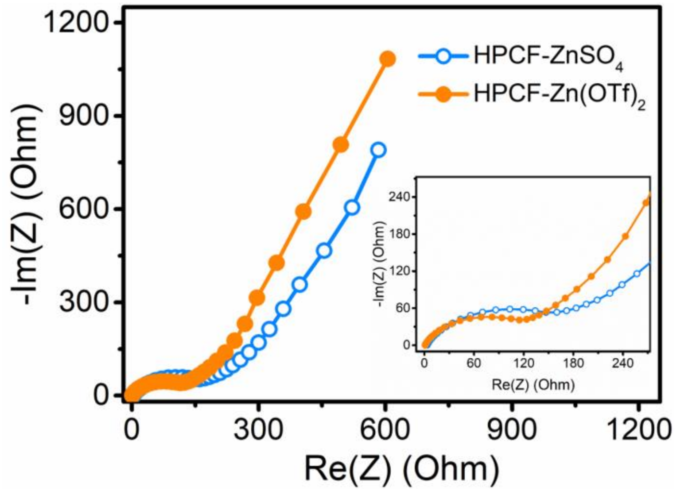

Fig. S13 EIS spectra of HPCF cathode in 2 M $ZnSO_4$ and 2 M $Zn(OTf)_2$ aqueous electrolytes

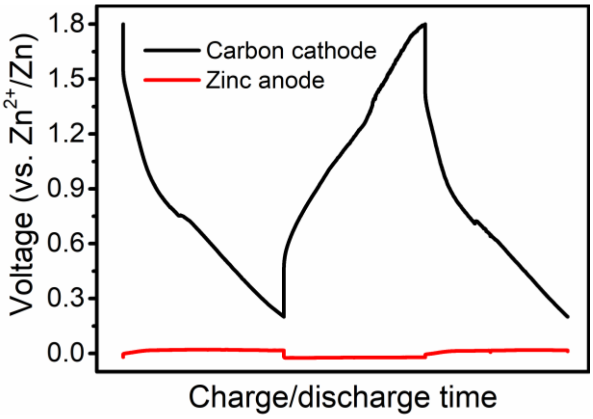

Fig. S14 Electrode potentials of carbon cathodes and zinc anodes during charge/discharge processes of ZHSs at ~0.1 A g^{-1}

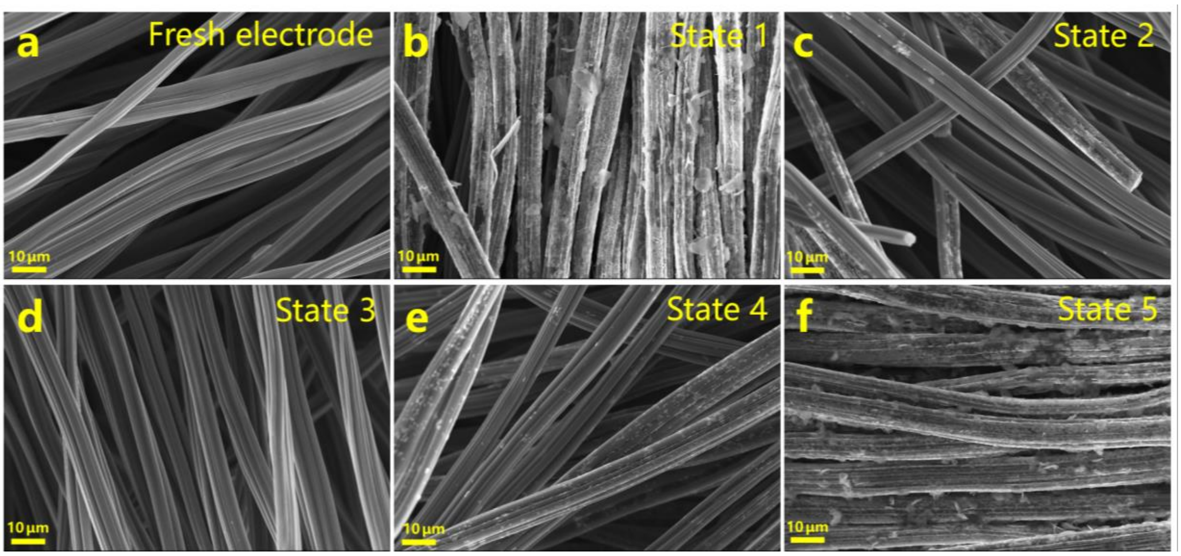

Fig. S15 SEM images at low magnifications of HPCF cathode at various charge/discharge states: (**a**) fresh cathode; (**b**) discharge to 0.2 V; (**c**) discharge to 0.2 V and then charge to 1.0 V; (**d**) discharge to 0.2 V and then charge to 1.8 V; (**e**) discharge to 0.2 V, then charge to 1.8 V, and finally discharge to 1.0 V; (**f**) discharge to 0.2 V, then charge to 1.8 V, and finally discharge to 0.2 V

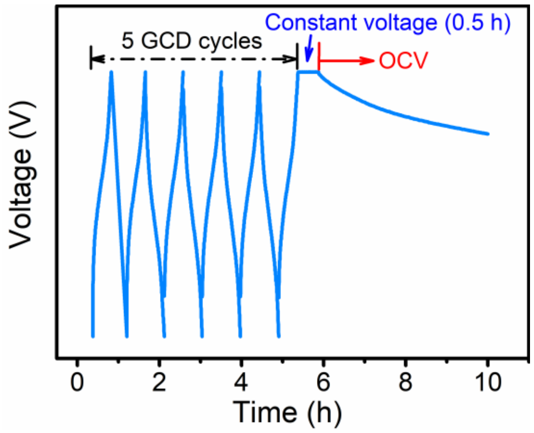

Fig. S16 Illustration of self-discharge behavior test. An electrochemical device such as ZHS, Zn-ion battery or supercapacitor was charged/discharged through GCD technique at 0.1 A g^{-1} for 5 cycles and then maintained at an expected voltage using constant voltage charging technique for 30 min. This is to make internal environment of the electrochemical device reaches a relatively stable state.

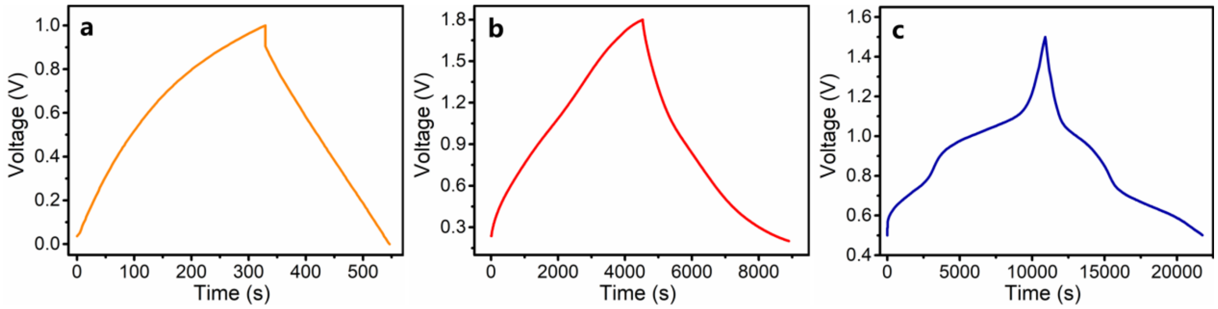

Fig. S17 GCD curves at 0.1 A g^{-1} of (**a**) AC//AC symmetric supercapacitor, (**b**) AC//Zn ZHS, and (**c**) $V_{10}O_{24}\cdot 12H_2O$//Zn Zn-ion battery. 2 M ZnSO4 aqueous electrolyte is used in the three electrochemical energy storage systems. AC materials with a high specific surface area (*e.g.*, 1923 m^2 g^{-1} for the used AC material herein) are representative electrode materials for symmetric supercapacitors and ZHSs,[S2] and vanadium oxides such as $V_{10}O_{24}\cdot 12H_2O$ are high-performance cathode materials for Zn-ion batteries [S3]. Therefore, AC//AC symmetric supercapacitor, AC//Zn ZHS and $V_{10}O_{24}\cdot 12H_2O$//Zn Zn-ion battery are chosen in this work and their self-discharge behaviors are compared with that of our fibrous carbon cathode-based ZHSs. Specific capacity of the AC//AC symmetric supercapacitor, AC//Zn ZHS and $V_{10}O_{24}\cdot 12H_2O$//Zn Zn-ion battery is 6, 121, and 300 mAh g^{-1}, respectively. More information about the synthesis, physicochemical characteristics and electrochemical performance of the used AC and $V_{10}O_{24}\cdot 12H_2O$ materials herein can be found in our previous work [S2, S3].

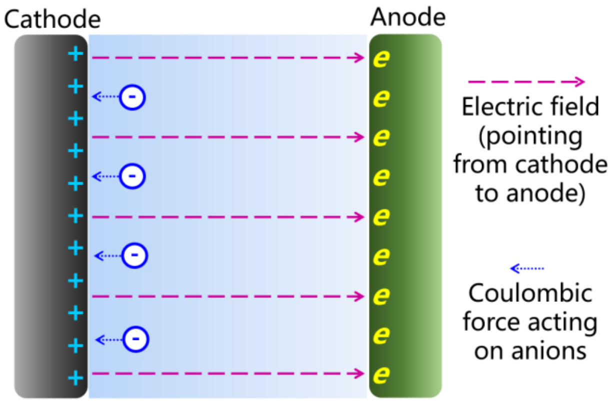

Fig. S18 Illustration of electric field and its effect on anions insides ZHSs. Movement of anions on cathode surface is restricted by coulombic force.

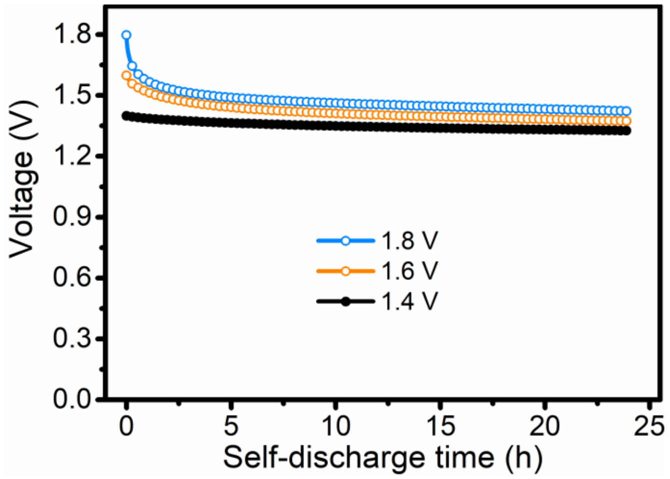

Fig. S19 Self-discharge behaviors of HPCF cathode-based ZHS with different initial voltages of 1.4, 1.6 and 1.8 V

Table S1 Summary of surface area and pore structure of the samples

Sample	BET surface area (m^2 g^{-1})	Pore volume (cm^3 g^{-1})		
		Micropore	Mesopore	Macropore
**CF**	10	0.001	0.005	~0
**MPCF**	810	0.310	0.026	0.003
**HPCF**	2000	0.581	0.279	0.021

Table S2 Energy-power density of ZHSs with various cathode materials [a]

Cathode	Electrolyte	Voltage window (V)	Capacity (mAh g^{-1})	Maximum energy density (Wh kg^{-1})	Maximum power density (kW kg^{-1})	*Refs.*
HPCF	$ZnSO_4$ (aq)	0.2-1.8	141 (0.1 A g^{-1})	112	14.5	This work
MPCF	$ZnSO_4$ (aq)	0.2-1.8	85 (0.1 A g^{-1})	65	3.7	This work
HPCF	$Zn(OTf)_2$ (aq)	0.2-1.8	141 (0.1 A g^{-1})	127	15.3	This work
MPCF	$Zn(OTf)_2$ (aq)	0.2-1.8	141 (0.1 A g^{-1})	72	10.1	This work
CF	$ZnSO_4$ (aq)	0.2-1.6	0.6 (0.1 A g^{-1})	0.4	0.6	This work
AC	$ZnSO_4$ (aq)	0.2-1.8	121 (0.1 A g^{-1})	84	14.9	[S2]
MCHS	$ZnSO_4$ (aq)	0.2-1.8	174.7 (0.1 A g^{-1})	220.1	11.9	[S4]
aMEGO	$Zn(OTf)_2$ (aq)	0-1.9	111.1 (0.1 A g^{-1})	106.3	31.4	[S5]
HPC	$ZnSO_4/Na_2SO_4$ (aq)	0-1.8	~200 (0.1 A g^{-1})	118	-	[S6]
N-HPC	$ZnSO_4$ (aq)	0.2-1.8	136.8 (0.1 A g^{-1})	191	3.63	[S7]
LDC	$ZnSO_4$ (gel)	0.2-1.8	127.7 (0.5 A g^{-1})	97.6	12.1	[S8]
HPC/CC	$ZnSO_4$ (aq)	0.2-1.8	138.5 (0.5 A g^{-1})	110	20	[S9]
Hydrous RuO_2	$Zn(OTf)_2$ (aq)	0.4-1.6	122 (0.1 A g^{-1})	82	16.74	[S10]
MXene	$ZnSO_4$ (aq)	0.01-1	60 (0.05 A g^{-1})	-	-	[S11]
MXene-rGO	$ZnSO_4$ (aq)	0.2-1.6	50 (0.4 A g^{-1})	34.9	0.28	[S12]

[a]Previously reported cathodes are composed of active materials, conductive additives, binder and current collectors, but their capacity, energy density and power density are calculated only based on active materials. Our fibrous carbon cathodes are free-standing electrodes. Therefore, if the whole mass of above cathodes were used to calculate capacity, energy

density and power density, our fibrous carbon cathodes will have a much bigger advantage over other cathodes.

Supplementary References

[S1] L. Dong, G. Liang, C. Xu, W. Liu, Z.Z. Pan et al., Multi hierarchical construction-induced superior capacitive performances of flexible electrodes for wearable energy storage. Nano Energy **34**, 242 (2017). https://doi.org/10.1016/j.nanoen.2017.02.031

[S2] L. Dong, X. Ma, Y. Li, L. Zhao, W. Liu et al., Extremely safe, high-rate and ultralong-life zinc-ion hybrid supercapacitors. Energy Storage Mater. **13**, 96 (2018). https://doi.org/10.1016/j.ensm.2018.01.003

[S3] W. Liu, L. Dong, B. Jiang, Y. Huang, X. Wang et al., Layered vanadium oxides with proton and zinc ion insertion for zinc ion batteries. Electrochim. Acta **320**, 134565 (2019). https://doi.org/10.1016/j.electacta.2019.134565

[S4] P. Liu, W. Liu, Y. Huang, P. Li, J. Yan et al., Mesoporous hollow carbon spheres boosted, integrated high performance aqueous Zn-Ion energy storage. Energy Storage Mater. **25**, 858 (2020). https://doi.org/10.1016/j.ensm.2019.09.004

[S5] S. Wu, Y. Chen, T. Jiao, J. Zhou, J. Cheng et al., An aqueous Zn-ion hybrid supercapacitor with high energy density and ultrastability up to 80 000 cycles. Adv. Energy Mater. **9**, 1902915 (2019). https://doi.org/10.1002/aenm.201902915

[S6] P. Yu, Y. Zeng, Y. Zeng, H. Dong, H. Hu et al., Achieving high-energy-density and ultra-stable zinc-ion hybrid supercapacitors by engineering hierarchical porous carbon architecture. Electrochim. Acta **327**, 134999 (2019). https://doi.org/10.1016/j.electacta.2019.134999

[S7] P. Liu, Y. Gao, Y. Tan, W. Liu, Y. Huang et al., Rational design of nitrogen doped hierarchical porous carbon for optimized zinc-ion hybrid supercapacitors. Nano Res. **12**, 2835 (2019). https://doi.org/10.1007/s12274-019-2521-6

[S8] Y. Lu, Z. Li, Z. Bai, H. Mi, C. Ji et al., High energy-power Zn-ion hybrid supercapacitors enabled by layered B/N co-doped carbon cathode. Nano Energy **66**, 104132 (2019). https://doi.org/10.1016/j.nanoen.2019.104132

[S9] X. Deng, J. Li, Z. Shan, J. Sha, L. Ma et al., A N, O co-doped hierarchical carbon cathode for high-performance Zn-ion hybrid supercapacitors with enhanced pseudocapacitance. J. Mater. Chem. A **8**, 11617 (2020). https://doi.org/10.1039/D0TA02770G

[S10] L. Dong, W. Yang, W. Yang, C. Wang, Y. Li et al., High-power and ultralong-life aqueous zinc-ion hybrid capacitors based on pseudocapacitive charge storage. Nano-Micro Lett. **11**, 94 (2019). https://doi.org/10.1007/s40820-019-0328-3

[S11] P.A. Maughan, N. Tapia-Ruiz, N. Bimbo, In-situ pillared MXene as a viable zinc-ion hybrid capacitor. Electrochim. Acta **341**, 136061 (2020). https://doi.org/10.1016/j.electacta.2020.136061

[S12] Q. Wang, S. Wang, X. Guo, L. Ruan, N. Wei et al., Adv. Electron. Mater. **5**, 1900537 (2019). https://doi.org/10.1002/aelm.201900537
